# Reimagining How We Treat Acute Pain: A Narrative Review

**DOI:** 10.7759/cureus.23992

**Published:** 2022-04-09

**Authors:** Pablo Castroman, Ovelio Quiroga, Victor Mayoral Rojals, Maria Gómez, Eleni Moka, Joseph Pergolizzi Jr, Giustino Varrassi

**Affiliations:** 1 Anesthesiology, University of Montevideo, Montevideo, URY; 2 Pain Medicine, Clinica del Dolor, Coquimbo, CHL; 3 Anesthesia, Belvitge Hospital, L'Hospitalet de Llobregat, ESP; 4 Pain Management, Federación Latinoamericana de Associaciones para el Estudio del Dolor, Hospital Universtario Nacional de Colombia, Bogatá, COL; 5 Anesthesiology, Creta Interclinic Hospital, Herakleion, GRC; 6 Anesthesiology, Nema Research, Inc, Naples, USA; 7 Pain Management, Paolo Procacci Foundation, Rome, ITA

**Keywords:** persistent postsurgical pain, multi-modal therapy, biopsychosocial factors, pain chronification, pain management, chronic pain, acute pain

## Abstract

Acute pain may be influenced by biopsychosocial factors. Conditioned pain modulation, distraction, peripheral nerve stimulation, and cryoneurolysis may be helpful in its treatment. New developments in opioids, such as opioids with bifunctional targets and oliceridine, may be particularly suited for acute pain care. Allosteric modulators can enhance receptor subtype selectivity, offering analgesia with fewer and/or less severe side effects. Neuroinflammation in acute pain is caused by direct insult to the central nervous system and is distinct from neuroinflammation in degenerative disorders. Pharmacologic agents targeting the neuroinflammatory process are limited at this time. Postoperative pain is a prevalent form of acute pain and must be recognized as a global public health challenge. This type of pain may be severe, impede rehabilitation, and is often under-treated. A subset of surgical patients develops chronic postsurgical pain. Acute pain is not just temporally limited pain that often resolves on its own. It is an important subject for further research as acute pain may transition into more damaging and debilitating chronic pain. Reimagining how we treat acute pain will help us better address this urgent unmet medical need.

## Introduction and background

Acute and chronic pain are often differentiated temporally with acute pain being of shorter duration, but their underlying mechanisms are fundamentally different. Acute pain occurs when noxious stimuli perceived by the peripheral nervous system are transmitted and along the way modulated by the central nervous system. On the other hand, chronic pain is associated with aberrant pain signal processing and interpretation and, as such, is much more challenging to treat. Cerebral neuroplasticity may cause maladaptive changes with persistent pain, inducing peripheral and central sensitization. Prompt and effective treatment of acute may interrupt potential pain chronification, which involves a complex interplay of ion channels, receptors, neurotransmitters, and neural systems [1]. Thus, the transition from acute to chronic pain involves a transition of the underlying pain mechanisms [2].

Pain intensity can be assessed using validated tools, but they offer only a unidimensional measurement of the pain; in reality, even acute pain can be complex and colored by biopsychosocial factors [3]. Acute pain is often is far more complex than previously realized and its effective treatment of far greater importance than previously realized. It is therefore time to reimagine how we treat acute pain. This is a narrative review based on proceedings from a conference in Lima, Peru, held in October of 2021.

## Review

It is difficult to overstate the global prevalence of acute pain, which is the main reason for up to 70% of visits to emergency departments [4]. Acute pain is a primary reason for visits to the doctor and many hospitalized patients experience acute pain during their stay [5]. Acute postsurgical pain, which occurs in about 80% of surgery patients, is a common form of acute pain and can be severe. Over a third (38%) of unexpected hospital readmissions following ambulatory surgery occur on account of pain [6].

Promising new treatment options for acute pain are emerging from translational pain medicine. The sensory experience of acute pain is surprisingly complex because the interactions between afferent inputs and their processing across the peripheral and central nervous systems involve affective-motivational components [7]. The acute pain experience is dynamic because it may be colored by a variety of fleeting and long-term psychological and psychosocial factors. The old stimulus-response view of acute pain is outmoded; in reality, pain signaling, like the experience of pain, is far more nuanced. The old theory that the experience of pain began in the thalamus of the brain has given way to the newer idea that the perception of pain arises in the cortex of the brain, albeit with certain thalamic inputs [8,9].

Drug development for analgesics is complex because there is no single main target for these agents; the most familiar of these analgesic categories target different mechanisms. Many analgesics developed fail to be cleared for market release because they either lack efficacy and/or present adverse safety profiles [10]. Opioids, cannabinoids, N-methyl-D-aspartate (NMDA) modulators, and serotonin modulators target the central nervous system; voltage-gated sodium channel (NaV) inhibitors, NMDA modulators, calcium modulators, and gamma-aminobutyric acid (GABA) modulators also target the dorsal horn; and nonsteroidal anti-inflammatory drugs (NSAIDs), NaV inhibitors, nerve growth factor (NGF) modulators, and transient receptor potential cation channel subfamily vanilloid (V) member-1 (TRPV1) modulators work on the peripheral nervous system (Table 1) [10].

**Table 1:** The main analgesic drug classes currently in development. GABA: gamma-aminobutyric acid; NGF: nerve growth factor; NMDA: N-methyl-D-aspartate; NSAID, nonsteroidal anti-inflammatory drug; TRPV1: transient receptor potential cation channel subfamily V member-1; NSAID: non-steroidal anti-inflammatory drug; NaV: voltage-gated sodium channel

Drug Class	Example	Comments
Opioids	Oxycodone, morphine	Includes new formulations, abuse-deterrent products, prodrugs
NSAID	Ibuprofen	Includes new formulations, delivery systems
Voltage-gated sodium channel (NaV) inhibitor	Lidocaine	Includes lidocaine patches (new delivery system) as well as mexiletine, moricizine, and others
Cannabinoid	Dronabinol	Promising preclinical results but mixed results in human pain syndromes
NMDA modulator	Ketamine	Modulates glutamates
NGF modulator	Tanezumab	Studies halted temporarily due to joint destruction
Serotonin modulator	Sumatriptan	There are 14 different 5-HT receptor subtypes
TRPV1 modulator	Capsaicin	Includes many “natural products” (e.g. chilli pepper)
Calcium modulator	Gabapentinoids	Also used as anticonvulsants
GABA modulator	Benzodiazepines	Not classic analgesics; currently have black-box warning in the United States

Nonpharmacologic options may not always be considered for acute pain, but distraction has emerged as a viable method to reduce acute pain by diverting the finite attentional resources of the brain away from painful stimulation [11]. Emerging from these studies of distraction are the ideas that learning mechanisms, such as pain catastrophizing, can exacerbate pain, while pleasant distractions may intervene and blunt pain. Distraction as pain control is not well studied, but appears to be more effective in pediatric than in adult patients [12,13].

Conditioned pain modulation (CPM) describes the decrease in perceived pain intensity for one stimulus after the application of a conditioning stimulus to another area of the body [14]. CPM has interpatient variability and is not present in some patients at all. In a study of 54 healthy adults, eight noxious heat stimuli were applied to the right side of the mouth while brain activity was evaluated using magnetic resonance imaging (MRI). After the first series, the process was repeated, but after the fourth stimulus, a separate noxious stimulus was used in the form of an intramuscular injection of hypertonic saline solution into the leg as a conditioning stimulus. CPM was observed in 23/54 patients as measured by reduced pain intensity [15]. In another study using cold pressor pain, 93% of healthy subjects exhibited clinically meaningful CPM [16]. CPM may be seen as a natural form of pain protection, but it is unclear why it exists only in a subset of the population [17]. Certain medications may interfere with CPM [18].

Transcranial direct-current stimulation (T-DCS), often used to treat chronic painful conditions, is now being studied in acute pain. In a study of 55 post-thoracotomy oncology patients who had intercostal nerve blocks and patient-controlled analgesia (PCA) morphine after surgery, patients who received anodal T-DCS over the left primary motor cortex for 20 minutes at 1.2 milliamperes (mA) for five consecutive days consumed significantly less postoperative morphine and had 80% less pain with cough five days after surgery than those patients who were administered sham stimulation. One year after surgery, both groups were similar with respect to the incidence of chronic pain and analgesic consumption [19]. In another study, T-DCS following thoracotomy was found to be a cost-effective method of pain control [20]. 

Peripheral nerve stimulation (PNS), used to treat chronic pain, is likewise being re-imagined for its possible role in treating acute pain. PNS requires the insertion of a lead under ultrasonic guidance, through which electric current flows to stimulate the nerve(s). PNS is not approved in some regions of the world, and evidence in support of its role is better established in chronic than acute pain [21]. Recently, ultrasound-guided percutaneous PNS has been shown effective to help manage postoperative pain [22]. In a study of five patients who underwent total knee arthroplasty, percutaneous PNS reduced pain by a mean of 93% at rest, and 80% of patients reported that their pain resolved completely [23]. 

Cryoneurolysis refers to the direct application of cold to a peripheral nerve, causing what has been called “reversible ablation,” meaning that it allows Wallerian degeneration and nerve regeneration to occur [24]. Cold-delivery wires called cryoprobes are placed percutaneously or through the surgical incision; a hand-held activator is available to initiate therapy delivery. Already used to treat the chronic condition of spasticity, cryoneurolysis has been shown effective in reducing acute postoperative pain [24].

Opioids have long been recognized for their role in acute pain control in appropriate patients. Oliceridine is a next-generation intravenous (IV) opioid that acts as a G-protein selective agonist at the µ-opioid receptor, allowing it to reduce the recruitment of β-arrestin, associated with adverse events [25]. In a randomized phase IIb study, 200 patients undergoing abdominoplasty were randomized to one of four groups: IV oliceridine with a loading dose of 1.5 mg and patient-controlled (PC) demand dosing of 0.10 (regimen A), IV oliceridine with a loading dose of 1.5 mg and PC demand doses of 0.35 mg (regimen B), IV morphine 4.0 mg loading dose and PC de9999mand doses of 1.0 mg (regimen C), and placebo. Treatment began four hours after surgery and continued for the next 24 hours. Both oliceridine groups provided significantly better pain control than placebo and offered pain relief similar to that of morphine, but with a significantly lower prevalence of adverse events [26]. Fewer opioid-associated adverse events and some early evidence that oliceridine presents a lower risk for opioid-induced respiratory depression make this a promising new agent for acute pain [27].

Bifunctional opioids are those that target more than one opioid receptor [28]. Opioids may bind with high affinity to κ, δ, and/or the nociception/orphanin FQ peptide receptors (NOP). In theory, the affinity for multiple receptors could enhance analgesia while reducing adverse effects [28]. Of these bifunctional opioids, the most attention has been devoted to a novel agent, KGNOP1, which has an affinity for both the µ- and NOP-receptors [29]. KGNOP1 is structurally based on a µ-opioid-receptor agonist and a weak nociception receptor antagonist (KGNOP3) that was developed to provide both nociceptive and neuropathic analgesia in a single agent [30]. In a murine study, KGNOP1 was effective in reducing experimental forms of both nociceptive and neuropathic pain [30]. By contrast, cebranopadol, a first-in-class analgesic currently under clinical investigation, is a full agonist at the NOP receptor [31,32]. 

Allosteric modulators are substances which, when binding to a particular receptor, change the receptor’s conformation and thereby increase or decrease its affinity for other molecules. For opioids, the recent discovery of specific allosteric ligands for G-protein-coupled receptors (GPCRs) may help with receptor subtype selectivity and could reduce potentially treatment-limiting opioid-associated side effects [33]. Positive allosteric modulators bind to sites on the µ-opioid-receptor different from the orthosteric site, where endogenous agonists bind. In the absence of the orthosteric agent, these positive allosteric modulators have only negligible effect, but they are capable of increasing or decreasing the potency of the orthosteric agonist [34]. Positive allosteric modulators are selective, and even related receptors of the same family may have different binding sites [35]. Allosteric agents targeting GPCRs allow for potentiation of allosteric modulation or negative allosteric modulation (NAM), but some Positive allosteric modulators also work with endogenous ligands. Moreover, there may be bitopic ligands that interact with both orthosteric and allosteric sites [36]. Allosteric modulation of GPCRs may be useful in the treatment of neurodegenerative disorders, such as Alzheimer’s disease and psychiatric illnesses such as schizophrenia [36]. Allosteric modulators have been found for µ-, δ-, and κ-opioid receptors, but no allosteric modulator has been found to date for NOP receptors. A study in a mouse model found the µ -opioid-receptor potentiation of allosteric modulation agent, BMS-986122, enhanced the ability of the endogenous opioid methionine-enkephalin to cause G-protein activity in the brain without any activity of its own, allowing greater inhibition of GABA release into the periaqueductal gray matter. The novel potentiation of allosteric modulation agent BMS-986122 does not bind to the orthosteric site and does not act as a µ-opioid-receptor agonist; for those reasons, it has fewer and milder side effects compared to conventional opioids [35,37].

Spahn and colleagues are investigating whether using the pathological conformation dynamics of opioid receptors - rather than physiological conformation - might affect the way opioid receptors interact with their ligands, specifically whether this interaction might occur without adverse effects [38]. Using computer simulations at low pH levels, they designed a selective agonist for the peripheral µ-opioid receptors near the site of injury and found this candidate agonist exhibited marked pH-sensitive binding in contrast to pharmaceutical fentanyl. In a murine study, this agent provided injury-specific analgesia without opioid-associated side effects [38].

The many voltage-gated ion channels in the body are transmembrane proteins that play important and varied roles. In particular, many subtypes of voltage-gated sodium (NaV) channels appear to be important in peripheral pain signaling. Loss-of-function mutations of NaV1.7 result in congenital insensitivity to pain, so NaV1.7 became an interesting new target for a pharmacologic antagonist [39]. Since such polymorphisms are rare, clinical investigative opportunities are limited. New data emerging suggest that any agent seeking to recapitulate the effects of a loss of function of NaV1.7 will have to penetrate the brain [39]. By the same token, gain-of-function mutations at NaV1.7 are associated with paroxysmal extreme pain disorder and other debilitating conditions [39]. Other NaV channels, namely NaV1.8 and NaV1.9, appear to play a role in somatic and visceral pain syndromes [39].

The role of transient receptor potential cation channel subfamily vanilloid (TRPV) member 1 (TRPV1) has been described as signal integration of molecular transmission from the nociceptors, thus playing a role in acute pain [40]. In a rat model of incisional pain, dilute capsaicin (0.025 to 0.10%) administered perineurally or via local infiltration reduced guarding behaviors and heat hyperalgesia following plantar incision with few and mild effects on mechanical responses [41]. Capsaicin was found to reduce calcitonin gene-related peptide (CGRP) and isoelectric BF/protein gene product 9.5-immunoreactivity of the nociceptors, thus making them less sensitive to noxious stimuli [41]. TRPV1-expressing fibers may be more important to pain signaling than the TRPV1 receptor itself [40].

Reimaging acute pain management also means repurposing established agents. Allopurinol, a xanthine oxidase inhibitor used in gout treatment, was compared to placebo in a study of postoperative pain following abdominal hysterectomy [42]. Fifty-four patients were randomized to receive 300 mg oral allopurinol or placebo the night before surgery and one hour immediately before the operation. Allopurinol was shown to significantly reduce postoperative pain by 40% two hours after surgery; anxiety scores after surgery were similar between groups [42]. Thus, the purinergic system may be a potential new drug target for analgesics treating acute pain.

Acute inflammation

Inflammatory pain is a protective response that can aid and encourage the healing of injured tissue. In a healthy individual, inflammation is managed by a complex cascade of pro-inflammatory and anti-inflammatory chemical mediators that balance each other [43]. Among them, cytokines play a key role by initiating and regulating inflammatory responses [43]. Inflammation occurs in response to trauma or other insult and should resolve as the underlying injury and tissue heal; however, there are cases where inflammation does not resolve with healing and becomes chronic and maladaptive. Inflammation may persist because of infiltration of myeloid-derived suppressor cells, excessive and prolonged inflammatory response, inadequate production of anti-inflammatory mediators, and other causes [44]. Chronic inflammation is associated with numerous pathologic conditions, including cancer, obesity, rheumatoid arthritis, asthma, chronic obstructive pulmonary disorder, multiple sclerosis, Crohn’s disease, ulcerative colitis, and others [44].

Neuroinflammation is a cascade of inflammatory responses triggered by neuronal activity and is characterized by the release of pro-inflammatory mediators, activation of glia, and infiltration of immune cells into both peripheral and central nervous systems [45]. Neuroinflammation is more likely to progress to chronic pain than systemic inflammation [46]. Typically, acute pain is associated with an acute inflammatory response with transient central sensitization, and this inflammation resolves over time. If it fails to resolve, it can result in long-term or even permanent central sensitization and chronic painful syndromes. Thus, a better understanding of neuroinflammation in the context of acute pain is needed. Nonsteroidal anti-inflammatory drugs (NSAIDs) are important agents for managing acute inflammatory pain because they inhibit cyclo-oxygenase-1 and/or -2 (COX-1 and -2) and prostaglandin production. Numerous NSAIDs are on the market, including selective COX-2 inhibitors (coxibs). Novel analgesics are in development. Among them are lipid mediators, such as resolvins, protectins, and lipoxygenase interaction products (lipoxins), which restore homeostasis to inflamed tissue.

There are specialized pro-resolving mediators that can aid in the clearance of tissue pathogens and debris around infectious inflammation [47]. For example, following spinal cord injury, anti-algesic lipid mediators are released to suppress inflammation. Their actions are mediated by GPCRs found on macrophages and glia [48]. Other novel agents for the control of inflammation include cannabinoids, which have been shown to produce antinociception in animals [49]. Humans have two cannabinoid receptors, known as CB-1 and CB-2, and the psychoactive effects of cannabis appear to be mediated entirely through CB-1. Both CB-1 and CB-2 appear to reduce inflammation [50]. However, the evidence for the use of cannabinoids for acute pain and inflammatory control remains mixed [51].

NSAIDs, particularly ibuprofen, diclofenac, and ketoprofen, are the most widely used analgesics for acute pain, but they have been associated with adverse events, such as gastrointestinal events or adverse cardiovascular events [52]. Long-term use of oral NSAIDs is not generally recommended [53]. Topical NSAIDs have provided good results in appropriate patients [45]. The short-term use of NSAIDs in appropriate patients may be helpful to manage acute pain.

Opioids can be effective pain relievers and are better suited for short courses than long-term use [45]. Pregabalin has been shown in preclinical trials to be effective against inflammatory pain and it is known to be effective in humans for treating neuropathic pain [45]. Corticosteroids may be used to address inflammatory pain and colchicine, which blocks IL-1β, may down-regulate multiple pathways for inflammatory pain [45].

Multimodal therapy uses a combination of agents with complementary mechanisms of action and often offers reduces opioid consumption without sacrificing analgesic benefits [45,54]. The ideal treatment for acute pain must first identify the cause of the pain and then based on an understanding of the underlying pain mechanism(s) and the molecular targets of various agents, arrive at a specific and precise analgesic regimen that is individualized to meet the needs of the patient [55].

Acute postoperative pain

Uncontrolled postoperative pain increases the risk of morbidity, dysfunction, decreased quality of life, impaired sleep, delayed recovery time, higher associated healthcare costs, and increased consumption of postoperative opioids [56]. Poorly controlled postsurgical pain can become chronic, resulting in persistent postsurgical pain (PPSP), which is far more challenging to treat [57]. In a study of 411 cognitively intact elderly patients undergoing surgery for hip fracture, higher pain scores at rest were significantly associated with longer hospital stays, physical therapy sessions, and delayed ambulation. At six months, those with higher pain scores at rest had significantly longer hospital stays, were more likely to miss physical therapy, and had lower locomotion scores both after surgery and at six months [58]. Postoperative pain at rest was associated with long-term functional impairment, although in this study it was not associated with residential care placement or six-month survival.

In a prospective survey of 625 elective surgery patients, patients were asked about pain prior to surgery, four days post-surgery, and again at six months [59]. Patients reporting high levels of pain four days after their operation (severe acute postoperative pain) and those whose surgeries lasted more than three hours were at significantly increased risk for increased pain, functional deficits, poor global recovery, and reduced quality of life at six months. There is a biopsychosocial aspect to this transition of acute to chronic pain because the fear of long-term consequences of surgery prior to the operation was associated with higher pain levels, poor global recovery, and worse quality of life after six months, whereas optimism prior to surgery was associated with improved recovery and better quality of life [59]. Yet biopsychosocial contexts for acute postoperative pain are not often considered in real-world clinical practice.

The type of surgery may also influence which patients develop PPSP. The incidence of PPSP is 30% to 50% for amputations and coronary artery bypass graft surgery; 30% to 40% for thoracotomy; 20% to 30% for breast surgery; and 10% for inguinal hernia or Cesarean section [57]. In a survey of 110 thoracotomy patients, 68% reported persistent pain three months after the operation, of whom 11% said pain levels >3 on a 0-10 scale and they reported significantly reduced function and vitality compared to those without pain [60]. PPSP is considered “severe” in 2% to 10% of these patients and often has a neuropathic component [57].

Since opioids are effective pain relievers, their short-term use to manage acute postoperative pain can benefit appropriate patients; current guidelines from the United States state opioids are to be used at the lowest effective dose for the shortest period of time [61]. There may be important considerations for patients who are currently taking opioids compared to those who do not take them at all. In a retrospective review of 6,364 patients who underwent unilateral total knee arthroplasty, it was found 24% were taking opioids prior to surgery, and of that group of opioid-experienced patients, 14% continued to take opioids 12 months after the procedure. Most patients in this study did not take opioids prior to the surgery and discontinued them within 12 months post-surgery. Of the opioid-naive patients who had not taken any opioids before surgery, only 3% were consuming opioid analgesics at 12 months. Thus, opioid-naïve patients were less likely to continue taking opioids than opioid-experienced patients. Furthermore, patients who took opioids 12 months following surgery were more likely to report greater pain at 12 months than those who did not continue taking opioids [62].

The differences between opioid-naïve and opioid-experienced surgical patients are telling. In a study of 574 patients undergoing either total knee or hip arthroplasty, 29.0% (n=167) were taking opioids on the day of their operation and experienced significantly greater pain at the surgical site, worse joint stiffness, more functional deficits, worse overall body pain, and significantly more symptoms of depression, anxiety, and catastrophizing compared to those who were not taking opioids prior to surgery [63]. Taking opioids before or after surgery exposed patients to the risk of prolonged opioid use, although, paradoxically, opioids still remain an important agent for the treatment of postoperative pain. While opioids are effective, they are also associated with adverse drug events which, in turn, were associated with higher costs and increased length of stay [64]. In a retrospective analysis of 592,127 surgical inpatients, opioids were associated with respiratory depression with rates ranging from 3% to 17% based on the type of surgery and incidences of nausea and vomiting ranging from 44% to 72% [65]. These complications could sometimes be associated with greater costs and longer hospital stays. Thus, the role of opioids in managing postoperative pain is coming under increasing scrutiny. 

It has been recommended that hospitals incorporate an acute pain service into their system to help better manage acute pain, particularly acute postsurgical pain. Despite greater awareness of PPSP and acute pain in general, the rates of PPSP have not decreased [66]. A general rule of thumb holds that about 10% of the surgical population will go on to have PPSP, which typically starts as acute pain and transitions into a chronic pain syndrome with a neuropathic component [2,45]. Such PPSP syndromes do not typically respond to opioid analgesia [67]. The neuropathic component of PPSP may pose challenges to treatment. In a systematic literature review, Haroutiunian and colleagues found that PPSP was likely to have a neuropathic component in 66% to 68% of breast cancer patients, 31% in patients undergoing groin hernia repair, and 6% after total hip or knee arthroplasty [68]. It is important for clinicians to recognize that PPSP can occur even after relatively minor surgical procedures, and it may develop in pediatric as well as adult or elderly patients. While only 2.2% of PPSP patients categorize their pain as severe, a neuropathic component may lead to more intense pain and greater dysfunction [69,70]. When diagnosing PPSP, it is important to rule out pre-existing painful conditions; postoperative complications, such as infection; and recurrence of the underlying disease or condition [71]. PPSP should also have lasted at least six months and be localized to the surgical field, innervation area of the nerve(s) in the surgical field, or the dermatome [72]. 

Patient factors may also contribute to acute pain. An observational study and survey of 100 patients who were diagnosed with post-traumatic and/or postsurgical neuropathic pain (mean pain levels 5.6 on a 0-10 scale) found that more severe pain was correlated with the number of comorbidities, and those with the most severe pain reported significantly more dysfunction, sleep problems, and depression compared to those with less-severe pain levels [73]. 

In a retrospective database study of 36,177 surgical patients (29,068 minor and 7,109 major surgery) who had filled a perioperative opioid prescription but had not taken opioids in the 12 months before the surgery, the rates of those with persistent opioid use were 5.9%, and 6.5% of minor and major surgery patients. In this study, persistent use was defined as refilling an opioid prescription between 90 and 180 days after surgery. The control group, who did not undergo surgery, had a much lower persistent opioid use rate of 0.4%. Some interesting patient risk factors for persistent opioid use emerged in this study: preoperative tobacco use, alcohol and/or substance use disorders, mood disorders, anxiety, and certain preoperative painful conditions, namely back pain, neck pain, arthritis, or centralized pain [74]. Since major surgery is likely to cause worse pain than minor surgery, but the prolonged use of opioids after surgery was similar for major and minor surgery patients, this suggests that protracted opioid use may be caused by reasons other than pain. This raises again the issue that patient factors and biopsychosocial considerations may be important for us to reframe acute pain and reimagine how it is treated. Genetic factors likely play an important role in terms of which surgical patients develop PPSP, but remain to be elucidated [75]. In addition to biopsychosocial factors, demographic information and clinical considerations relating to the type of surgery and underlying disease appear to play a part as well [76].

## Conclusions

Elucidation of the physiopathology of acute pain has resulted in new findings, namely that acute pain (like chronic pain) is a biopsychosocial phenomenon that may be influenced by numerous patient-specific and treatment-specific factors. As acute pain is elucidated, emerging treatments for it including nonpharmacological methods should be investigated. Conditioned pain modulation may be a pain-regulating technique for acute pain and other approaches such as PNS are being used for acute pain. New agents such as allosteric modulators may revolutionize opioid analgesia by modulating the response of opioid receptors. Opioids are effective analgesics but may in some cases paradoxically be associated with worse or more prolonged pain. Most acute painful symptoms involve an inflammatory component, but the inflammatory response in acute pain syndromes differs from chronic inflammation such as occurs in neurodegenerative disorders. Reimagining acute pain management means considering pain in context, evaluating novel agents and nonpharmacologic treatments, and being more cognizant of the prevalence of untreated or under-treated acute pain. In some cases, acute pain can transition into chronic painful conditions which are difficult to treat and may lead to dysfunction and disability.
